# Supplementary material for: Influences of Maternal Weight and Geographic Factors on Offspring Traits of the Edible Dormouse in the NE of the Iberian Peninsula
Source: Life (Basel). 2023 May 21;13(5):1223. doi: 10.3390/life13051223 (PMC10221900; doi:10.3390/life13051223)
Supplement: Supplementary file 1 [file life-13-01223-s001.zip › life-2369063-supplementary.pdf]

Article

Influences of maternal weight and geographic factors on offspring traits of the edible dormouse in the NE of the Iberian Peninsula

Silvia Míguez [1,*], Ignasi Torre [2], Antoni Arrizabalaga [2] and Lídia Freixas [2,*]

[1] Independent Researcher, E-08016 Barcelona, Spain
[2] BiBio Research Group, Natural Sciences Museum of Granollers, C/Francesc Macià 51, E-08402 Granollers, Spain; itorre@mcng.cat (I.T.)
* Correspondence: silvia.miguez.marcos@gmail.com (S.M.); lfreixas@mcng.cat (L.F.)

Supplementary S1

Mean litter size, body weight of pups by age group, and differences in offspring weight by sex and age.

This supplement contains field data collection details, information about the data preparation and data cleaning process prior to analysis, as well as tests to verify the assumptions of normality and equality of variance of the data.

Data used in these analyses have been collected over the period 2004-2021. To describe the mean litter size of the edible dormouse populations in Catalonia (NE Spain), pups with open eyes were excluded to minimize bias caused by litter sizes that do not contain the actual number of pups at birth, because young dormice are more vulnerable during the third stage (22-30 days) due to the higher probability of predation [76]. Therefore, only the first two age groups (i.e., pink pups and gray pups) were used. Duplicate data from females captured with pups more than once in the same year were eliminated, giving priority in this case to litter sizes with pink pups. Litter records with dead pups or live pups without the presence of the mother were also not used. The final dataset (n = 131) included litter sizes from 20 sampling stations in six natural areas from Catalonia. Offspring mean weight according to the three age groups (pink pups, gray pups, and open eyes pups) and sex was reported. The initial dataset contained 1170 offspring weights and was utilized to describe the average weights according to the age. Pups whose data were incomplete (e.g., missing weight) and weights of dead pups were not included. Furthermore, the weights corresponding to live pups that were found without the mother (e.g., abandoned because the mother was predated, or other causes) were also excluded from the investigation due to their weights being lower than normal weight because they were not suckled for some unknown time. An exception was only made in cases of communal nests in which one of the mothers was not present, assuming that those pups were cared for and suckled by the other mother. Results were summarized in Table S1, Table S2 and Figure S1. A data subset was created (n = 1092) to evaluate differences in offspring weights by sex at each age group by removing unsexed pups. To ensure statistical independence, cases in which the same pups were weighed two times within the same age group were also excluded. Duplicate records of pups weighed in different ages were not eliminated because separate tests were performed for each age. Prior to statistical analysis, the assumptions of normality and homoscedasticity of the data were checked using Lilliefors-Corrected Kolmogorov-Smirnov test and Levene test, respectively. The *lillie.test* function in the *nortest* package [140] and the *leveneTest* function of the *car* package [141] were used to perform these tests (**Table S3**). Non-parametric Mann Whitney U-Test was performed to examine

differences in offspring weights by sex in each age group because the data were not normally distributed (Lilliefors-Corrected Kolmogorov-Smirnov test: $p < 0.05$) and there were some outliers in one of the age groups that were not removed from the analysis due to being real values (i.e., they contain valuable information about the offspring under investigation). Therefore, their inclusion gives a more accurate result despite increasing the variance. Specifically, separate Mann-Whitney U tests were performed in each age using the *wilcox.test* function in the *stats* package [77]. Results are summarized in the following table.

Table S1. Means, standard deviations and ranges for offspring weight of the edible dormouse at three age groups.

Pup age group	N	Mean[1] ± SD	Range (Min-Max)
Pink pups	140	4.82 ± 1.13	2.5-6.8
Grey pups	579	11.66 ± 3.88	4.0-25.5
Open eyes pups	451	23.60 ± 6.16	10.1-39.8

[1] Mean body weight (g).
Abbreviations: *N*, sample size; *SD*, standard deviation; *Min*, minimum; *Max*, maximum.

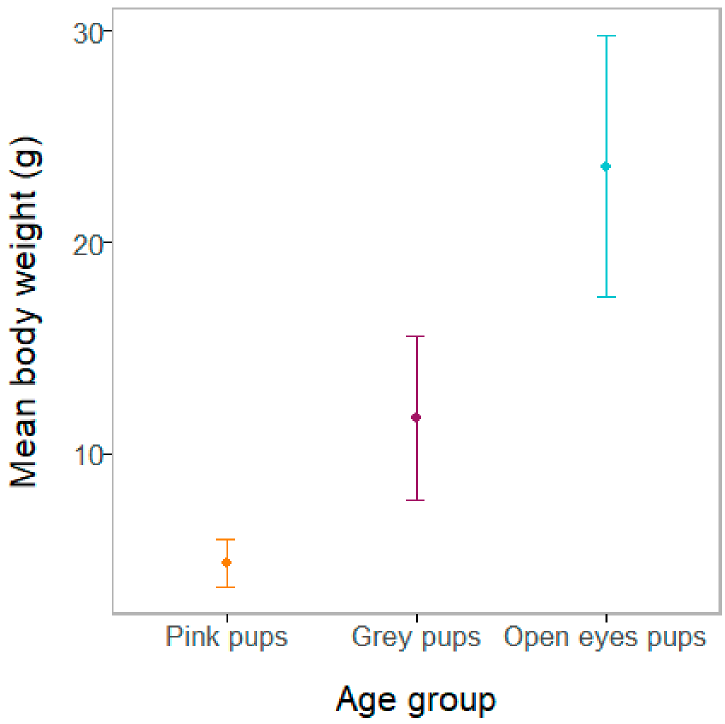

Figure S1. Averaged body weight (±SD) of the pups according to age groups.

Table S2. Summary statistics of pup weights by sex in the three age categories considered (pink pups, grey pups and open eyes pups) and results of the Mann-Whitney test to assess whether weights of the pups in each age group differed between sexes.

Pup age group	Sex	N	Mean[1] ± SD (range)	Mann-Whitney test
Pink pups	Female	51	4.79 ± 1.02 (2.7-6.8)	W = 1633.5, $p = 0.1659$
	Male	75	4.90 ± 1.16 (2.5-6.5)	
Grey pups	Female	238	11.24 ± 3.70 (4.0-24.7)	W = 32157, $p = 0.0552$
	Male	299	11.90 ± 3.92 (5.5-25.5)	

Open eyes pups	Female	195	23.49± 6.41 (10.1-39.8)	W = 23149, p = 0.7942
	Male	234	23.21 ± 5.72 (12.6-38.5)	

[1] Mean body weight (g).
Abbreviations: *N*, sample size; *SD*, standard deviation; *W*, statistic; *p*, *p*-value.
Null hypothesis is accepted if $p > 0.05$ and rejected if $p < 0.05$.

Table S3. Results of the Lilliefors-Corrected Kolmogorov-Smirnov test to assess normality and Levene's test to assess the equality of variances for offspring weight by sex in each age group.

	K-S with Lilliefors Correction Test		Levene's Test of Equality of Variances			
	Statistic	*p*	Statistic	df1	df2	*p*
**Pink pups**						
Males	0.17574	**5.084e-06**	1.4987	1	124	0.2232
Females	0.15162	**0.005036**				
**Grey pups**						
Males	0.084721	**2.166e-05**	1.158	1	535	0.2824
Females	0.084816	**0.0002757**				
**Open eyes pups**						
Males	0.080684	**0.0008374**	3.2498	1	427	0.07214
Females	0.089838	**0.0006017**				

Abbreviations: *K-S*, Kolmogorov-Smirnov; *df*, degrees of freedom; *p*, *p*-value.

Note: $p < 0.05$ are marked in bold face. Null hypothesis is accepted if $p > 0.05$ and rejected if $p < 0.05$.

Supplementary S2

Trade-Off between offspring number and size and effect of maternal body weight on mean pup weight.

This supplement provides an extensive description of the data obtained in the field and contains details of data preparation and cleaning prior to analysis. It also includes preliminary statistics such as saturated models, analyses to detect collinearity problems between predictor variables, and plots and tests to check the assumptions of the residuals in the selected linear regression models.

The main objective of this section was investigating the relationships between litter size and maternal body weight with mean pup weight at birth, as well as the correlation between litter size and mother's weight. Litter size (LS) was defined as the value of the total number of pups born per litter, maternal body weight (MBW) took the value of body weight of the mothers at the time that they were captured with pink pups (postpartum weight) and finally, mean pup weight (mPW) was the average weight for a pup per litter. In order to investigate these associations, the field research addressed two essential problems: (1) not always could be obtained offspring weight data when pups are newborn (e.g., first day of life) and (2) frequently, it was not possible to have the weight of the mothers before pregnancy (pre-pregnancy weight) or during gestation

to assess whether female pre-reproductive or gestational body mass were good predictors of birth weight in this species. Therefore, to reduce biases both in offspring weight change due to lactation and in the number of pups caused by mortality in older age classes [76,78], only data from very young offspring (i.e., pink pups, having less than 8 days old) were considered. In the case of females, the study used maternal body weight after partum as an approximation of the pre-pregnancy and/or gestational weight. To obtain the final dataset, litters with some dead pink pup, cases where the female escaped and their identity was not available, or litters that were found without their mothers, were excluded from the analysis.

Correlation analyses were carried out using *cor.test* function in *stats* package [77]. Ordinary Least Squares (OLS) linear regressions were conducted using the *lm* function in *stats* package [77] to explain the relationship between mPW (response variable) and LS and MBW as independent variables. First, the linear regression model including all predictors (and their interactions) was fitted to account for the potential masking effect of female size on the trade-off between offspring mass and number, but the results did not reveal significant interaction between MBW and LS (**Table S4**). No collinearity problems were detected for the model without interaction because correlation between predictors was $|r| < 0.5$ and all variance inflation factor (VIF) values were < 1.5 (**Table S5, Table S6**, **Figure S2**). Because two candidate models were found during model selection (i.e., ΔAICc < 2), and models with ΔAICc less than 2 are considered equivalent and as good as the best model [90,142], model averaging was used to average the parameter estimates of these two models and thus avoid model uncertainty [90,142]. The residual analysis indicated that the linear regression assumptions were satisfied for both models (**TableS7**, **Figure S3**).

Table S4. Summary of regression analysis with interaction between maternal body weight and litter size variables explaining mean pup weight of the edible dormouse population in Catalonia (Spain).

Response, model formula, model parameter	Estimate	Standard Error	*p*-value
**Mean pup weight:**			
mPW ~ LS * MBW			
(Intercept)	6.022	5.009	0.238
LS	-0.872	0.918	0.349
MBW	-0.022	0.049	0.650
LS x MBW	0.0099	0.009	0.272

Abbreviations: *mPW*, mean pup weight; *LS*, litter size; *MBW*, maternal body weight.
Note: $p < 0.05$ are marked in bold face.

Table S5. Summary of Pearson's (r) and Spearman's (r_s) correlation coefficients for the variables litter size, maternal body weight and mean pup weight of the edible dormouse population in Catalonia (Spain).

	Pearson	Spearman

	r	*p*	r_s	*p*
Litter size - Maternal body weight	0.23	0.1812	0.15	0.3898
Mean pup weight - Maternal body weight	0.43	**0.0098**	0.33	0.0504
Mean pup weight - Litter size	0.27	0.1123	0.31	0.0654

Note: $p < 0.05$ are marked in bold face.

Table S6. Summary of the results of variance inflation factor (VIF) and the tolerance value for collinearity check in the model without interaction evaluating the effects of litter size and maternal body weight on mean pup weight of the edible dormouse population in Catalonia (Spain).

**Response, model formula, model parameter**	**VIF**	**Tolerance**
**Mean pup weight:**		
mPW ~ LS + MBW		
LS	1.05	0.95
MBW	1.05	0.95

Abbreviations: *mPW*, mean pup weight; *LS*, litter size; *MBW*, maternal body weight. Predictors with VIF greater than 5 to 10 and tolerance lower than 0.1 to 0.2 would be considered as problematic.

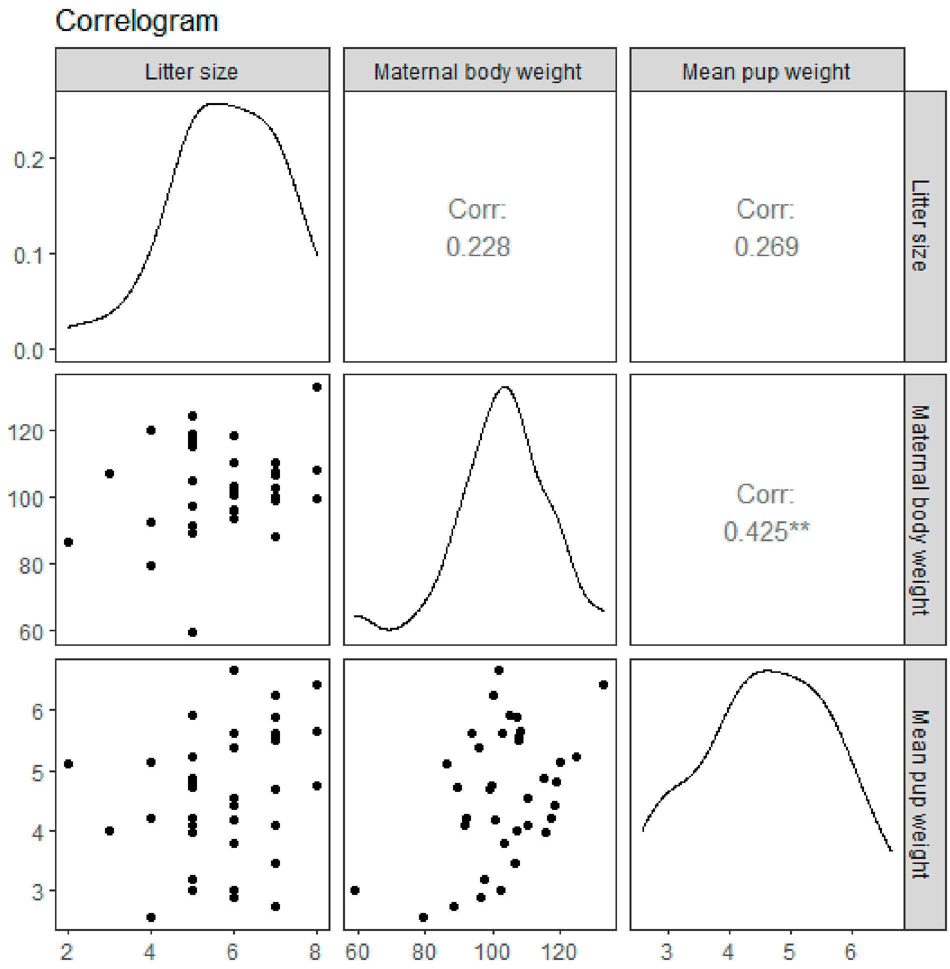

Figure S2. Correlation matrix plot for variables used in fitting models investigating the effects of litter size and maternal body weight on mean pup weight. The correlation is visualized as a scatterplot. The diagonal represents the distribution of each variable with a density plot. The Pearson's correlation coefficients indicate the strength and direction of the association between pairs of variables and the value obtained appears in the corresponding cell. The asterisk in the cell represents a statistically significant correlation (* $p < 0.05$, ** $p < 0.01$, *** $p < 0.001$).

Table S7. Results of the Shapiro-Wilk (SW), Breusch-Pagan (BP) and Durbin-Watson (DW) tests applied to residuals of the two best-fitted linear regression models to check the assumptions of normality, homoscedasticity, and autocorrelation of the residuals, respectively.

**Model formula**	**SW**		**BP**		**DW**	
	**Statistic**	***p*-value**	**Statistic**	***p*-value**	**Statistic**	***p*-value**
mPW ~ MBW	0.96249	0.2562	0.8149	0.3667	2.0404	0.5418
mPW ~ LS + MBW	0.97651	0.6271	2.0441	0.3599	2.0816	0.594

Abbreviations: *mPW*, mean pup weight; *LS*, litter size; *MBW*, maternal body weight.
Note: $p < 0.05$ are marked in bold face. Null hypothesis is accepted if $p > 0.05$ and rejected if $p < 0.05$. The DW statistic with a value near 2 indicates that no autocorrelation was detected.

a) Linear relationship (Top Left); Homogeneity of Variance (Top Right); Influential observations (Middle Left); QQ Plot (Middle Right); Density Plot (Bottom Left).

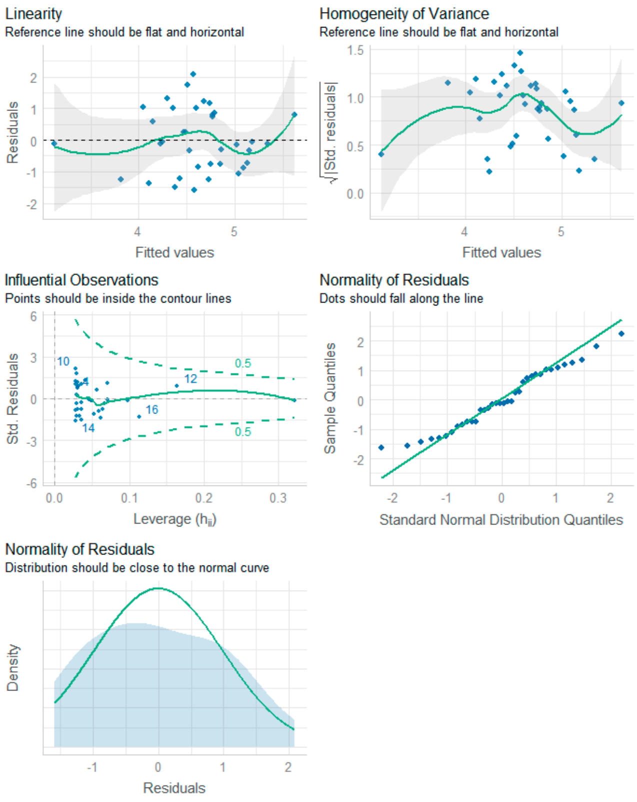

b) Linear relationship (Top Left); Homogeneity of Variance (Top Right); Variance Inflation Factor (VIF) (Middle Left); Influential observations (Middle Right); QQ Plot (Bottom Left); Density Plot (Bottom Right).

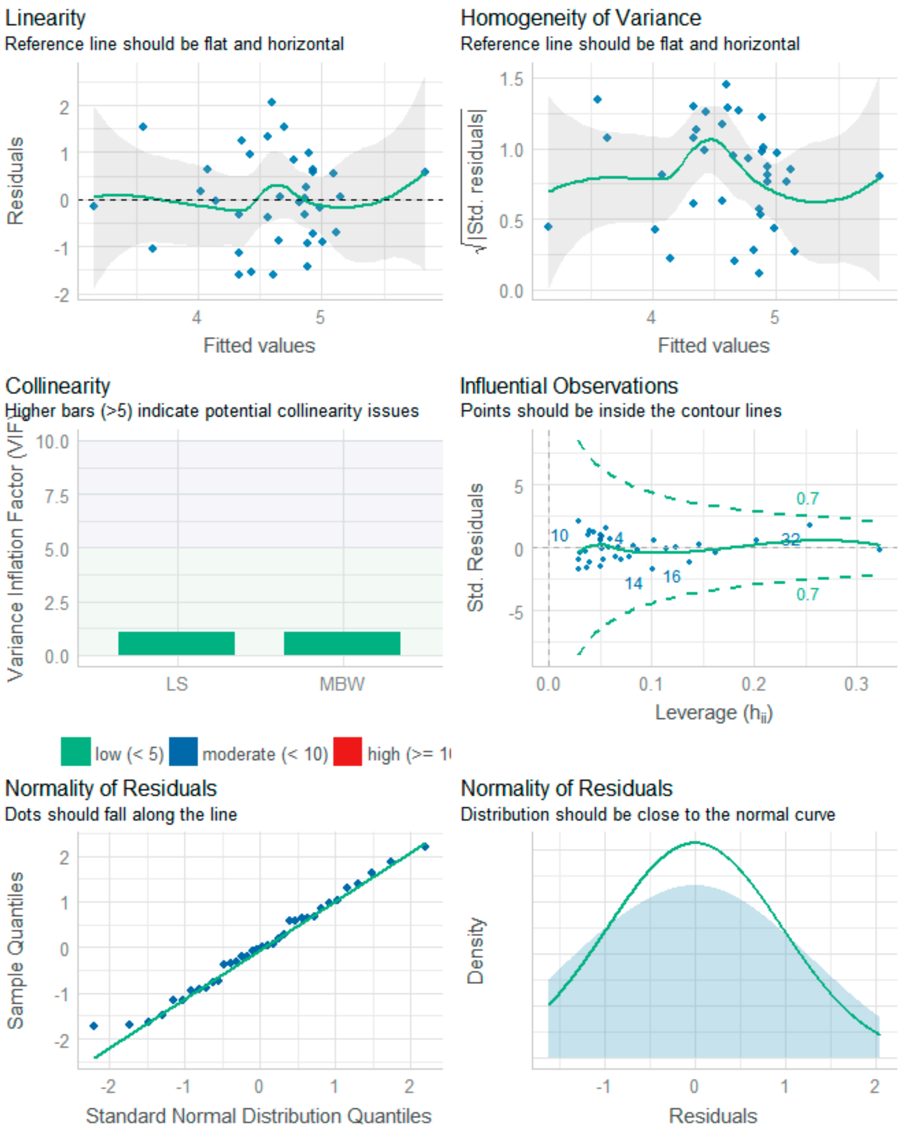

Figure S3. Diagnostics plots for the two best models selected based on ΔAICc < 2 in the linear regression analysis of mean pup weight at birth in edible dormouse (for more details, see **Table 2** in the *Results* section). In the two models evaluated, mean pup weight was the response variable, and the explanatory variables were (a) maternal body weight (MBW) and (b) litter size (LS) and maternal body weight (MBW).

Supplementary S3

Geographic patterns in litter size.

This supplement contains details regarding data obtained in the field, data preparation, and data cleaning prior to analysis. It also presents an overview of random effects structure (justified by the experimental design) utilized in the Generalized Linear Mixed Models fitted, and how were they computated. In addition, overdispersion tests, summary of Pearson's and Spearman's correlations coefficients between geographic explanatory variables and also between response variable (litter size) and predictors, summary of VIF and tolerance indices to detect collinearity problems, results of model selection based on AICc, and the results of principal component analysis (PCA) were also included.

The aim of this analysis was assessing the effects of geographic variables (latitude, longitude and elevation) in the litter size of the edible dormouse. The dataset used contains data from all nest boxes with presence of pups. Furthermore, data from the two sampling techniques (line transect method and plot method) were included to increase the available geographic gradient. For the same reason argued in *Materials and methods*, *section 2.2.1.*, only litters with pink and grey pups were used, and litters with open eyes pups were excluded. The geographic coordinates (decimal degrees) and elevation (m a.s.l.) of each nest box with pups were registered by means of the Global Positioning System (GPS). Altitudinal and latitudinal gradients share certain similarities with respect to the observed environmental transitions, such as temperature [143]. On the other hand, altitudinal gradients are not subject to the variation in seasonal changes in photoperiod that occurs across latitudes [130].

First, a Generalized Linear Mixed Model (GLMM) with a Poisson distribution and log link function [79] was run using the *glmmTMB* function in the *glmmTMB* package [80]. Litter size was response variable, and latitude, longitude and elevation were the explanatory variables. *Sampling station*, *Nest box identity*, *Female identity* and *Year* were included as crossed random factors to account for repeated measures of the same female in different years or sampling the same site and nest box repeatedly. The inclusion of these random effects will control for unmeasured differences between sampling sites and possible annual differences in climatic conditions and food availability. Because not all sampling stations were sampled during the same years, some sampling stations may be represented by a unique combination of years, and several years contains different combinations of stations. Furthermore, not all individuals (females with offspring) were found every year. That is, dataset presents litter size data from several females sampled different number of times across multiple years. The random effect is nested when a lower-level factor appears only within one of the determined level of an upper-level factor. In this analysis, there is a totally nested design for the factors concerning sampling site and females, as shown below: (1) Nest boxes are nested within Stations and (2) females are also fully nested within Stations. In contrast, some females appear in the same nest boxes in different years, but others occupy different boxes. In this case, females were considered partially crossed with nest box identity. The decision to introduce a fully crossed design for all random effects in the fitted mixed models instead of the requiered nested random factors was based on the complexity of the experimental design. In terms of computational considerations, the use of an implicit nesting (i.e., the nesting was previously defined in the dataset, where each identification code for nest boxes is unique in a given sampling station in a Natural Park and cannot refer to a nest box at any other station) allows the use of a crossed syntax rather than a nested syntax because in this case, R will give identical results. Before GLMMs analysis, all explanatory variables were centered and scaled to have mean of 0 and standard deviation of 1 using the *scale* function [77] to allow model convergence. Mixed-effects Poisson regression was evaluated to detect possible over- or underdispersion. Tests to verify the assumption that the conditional mean and variance are equal for the Poisson models were performed by means of overdispersion tests using *check_overdispersion* function in the *performance* package [87], and the results showed underdispersion (**Table S8**). Reproduction of individuals is typically recorded as count data (e.g., number of fledglings from a nest or inflorescences on a plant) and is commonly modeled using Poisson or negative binomial distributions, which assume that the variance is greater than or equal to the mean. However, the distributions of reproductive effort are often underdispersed (i.e., variance < mean). When used in hypothesis tests, models that ignore underdispersion will be overly conservative and may fail to detect significant patterns. Generalized Poisson (GP) and Conway-Maxwell-Poisson (CMP) distributions are better choices for modeling reproductive

effort because they can handle both overdispersion and underdispersion [144]. Hence, the model was refitted using generalized Poisson and/or Conway-Maxwell-Poisson distributions with a log link function. Model with the generalized Poisson distribution clearly resulted the most parsimonious and was selected (**Table S9**). Overall, the cross-correlations between the explanatory variables showed moderate to strong correlation coefficients that were statistically significant (**Table S10, Figure S4**). In some cases, variance inflation factor (VIF) values > 5 and tolerance indices < 0.2 also evidenced possible collinearity problems (**Table S11, Figure S5**). Second, to resolve the effects of multicollinearity among the set of geographic variables, Principal Component Analysis (PCA) was performed. Singular value decomposition (SVD) was the method used to perform principal component analysis (PCA) using the *prcomp* function in *stats* package [77]. The data were standardized to a mean of zero and standard deviation of one prior to running the PCA. Kaiser's rule was the method used to select the number of components in the PCA, maintaining the components with eigenvalues greater than 1 [81] (**Figure S6**). PCA plot was generated using the *fviz_pca_var* function in the *factoextra* package [145] (**Figure S7**). The PCA showed that 98.2% of the variability was explained by the first two principal components (PC1-PC2). The total variance explained by the first principal component (PC1) was 71.5% and was positively associated with latitude and negatively with longitude. The second principal component (PC2) accounted for 26.7% of the variance and was positively correlated mainly with elevation. Finally, the percentage of variation explained by the third component (PC3) was only 1.8% and was positively associated with latitude and longitude. According to Kaiser criterion, only the first component was considered (eigenvalue = 2.14) was considered, resulting in a single principal component important to interpret the geographic gradients (**Table S12**, **Figure S6**). Third, a new set of GLMMs were re-run following the steps described above, employing the scores of the first principal component (PC1) selected as the explanatory variable. In the fitted Poisson GLMM , underdispersion appeared again (**Table S8**). The final model selected for reporting in this study was GLMM fitted to generalized Poisson distribution and log link function, with litter size as the response variable and PC1 scores as the explanatory variable (**Table 4** in main text). Verification of equidispersion was performed by means of a nonparametric dispersion test through sd of residuals fitted vs simulated using the *testDispersion* function on *DHARMa* package [93] (**Table S13**).

Table S8. Table showing the results of overdispersion tests of GLMMs fitted to the Poisson distribution for the relationship between litter size (response variable) and latitude, longitude and elevation or first principal component (PC1) scores as explanatory variables. For both models, *Station*, *Nest box identity*, *Female identity*, and *Year* were included as crossed random effects.

**Model**	**Explanatory variables** *	**Overdispersion test**		
		X^2	**Dispersion ratio**	***p*-value**
Poisson	LAT<br>LON<br>ELEV	61.100	0.481	1
Poisson	PC1	62.412	0.484	1

Abbreviations: *LAT:* Latitude; *LON:* Longitude; *ELEV*: Elevation; PC1, scores of the first principal component of the PCA.

Note: $p < 0.05$ are marked in bold face. Equidispersion occurs if the dispersion parameter approaches 1.

* centered and scaled predictors to mean 0 and standard deviation 1.

Table S9. Model selection based on Akaike's Information Criterion corrected for small sample size (AICc) for generalized linear mixed models (GLMMs) examining the effect of standardized geographic variables (latitude, longitude and elevation) on litter size variation of edible dormouse populations across the NE of the Iberian Peninsula. Models are ranked according to their AICc in descending order of support.

Model	df	LogLik	AICc	ΔAICc	w_i
**Generalized Poisson model**	**9**	**-253.380**	**526.2**	**0.00**	**1.000**
Poisson model	8	-271.511	560.2	33.96	0.000

Abbreviations: *LogLik*, log-likelihood; *df*, degrees of freedom; *AICc*, Akaike's Information Criterion corrected for small sample sizes; *ΔAICc*, Delta AICc; w_i, Akaike's weight. Models are ranked in descending order of support, and models in bold ($\Delta AIC_c < 2$) having the greatest support in the data.

Table S10. The table shows Pearson's (r) and Spearman's (r_s) correlation coefficients between the geographic variables (independent variables), as well as the pairwise correlations between geographic variables with the response variable (litter size).

	Pearson		Spearman	
	**r**	***p***	**r_s**	***p***
Latitude - Longitude	-0.92	**< 0.001**	-0.67	**< 0.001**
Latitude - Elevation	0.46	**< 0.001**	0.75	**< 0.001**
Longitude - Elevation	-0.26	**0.0027**	-0.61	**< 0.001**
Litter size - Latitude	0.11	0.201	0.05	0.566
Litter size - Longitude	-0.12	0.176	0.01	0.925
Litter size - Elevation	-0.05	0.562	-0.08	0.346

Note: $p < 0.05$ are marked in bold face.

Table S11. Summary of the results of variance inflation factor (VIF) and the tolerance value for testing collinearity in final selected model (GLMM fitted to Generalized Poisson distribution and log link function) evaluating the effects of latitude, longitude, and elevation on litter size in the edible dormouse populations in the NE of the Iberian Peninsula.

Response, model formula, model parameter	VIF	Tolerance
**Litter size:**		
LS ~ LAT + LON + ELEV		
LAT *	11.16	0.09
LON *	9.61	0.10
ELEV *	1.63	0.61

Abbreviations: *LS*, litter size; *LAT:* Latitude; *LON:* Longitude; *ELEV*: Elevation.

Note: Predictors with VIF greater than 5 to 10 and tolerance lower than 0.1 to 0.2 would be considered as problematic. Random structure is omitted.
*centered and scaled predictors to mean 0 and standard deviation 1.

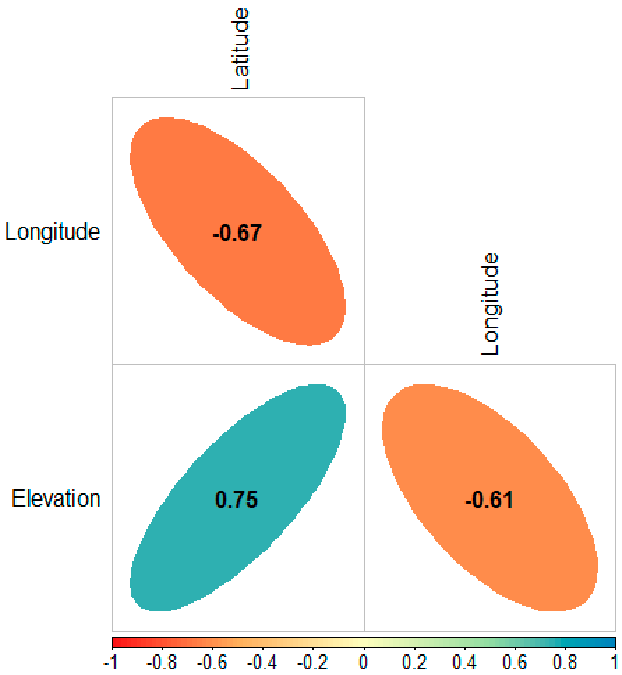

Figure S4. Geographic variables correlations. Spearman's rank correlation coefficient-based correlograms of the geographic predictors. The color of each ellipse indicates the value of the correlation coefficient for each pair of variables following the color scale of the horizontal color bar.

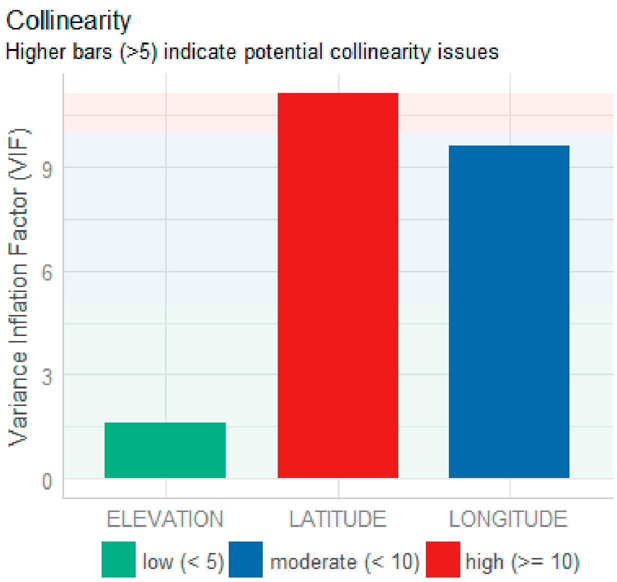

Figure S5. Bar plot showing the variance inflation factor (VIF) values for the collinearity check in final selected model (GLMM fitted to Generalized Poisson distribution and log link function) assessing the effects of latitude, longitude and elevation on litter size in the edible dormouse. Colors in the bars represent low collinearity (green), moderate collinearity (blue) and high collinearity (red).

Table S12. Summary of principal component analysis (PCA) statistics and eigenvectors of the three principal components (PC1-PC3) calculated from geographic variables data obtained for each nest box with presence of edible dormouse pups across the NE of the Iberian Peninsula. The geographic predictors are latitude (decimal degrees), longitude (decimal degrees) and elevation (m a.s.l.).

	**PC1**	**PC2**	**PC3**
Eigenvalue	2.144	0.801	0.054
Standard deviation	1.464	0.895	0.233
Proportion of total variance explained	0.715	0.267	0.018
Cumulative proportion of variance	0.715	0.982	1.000
Eigenvectors			
Latitude	0.666	- 0.161	0.729
Longitude	-0.626	0.411	0.663
Elevation	0.406	0.898	-0.173

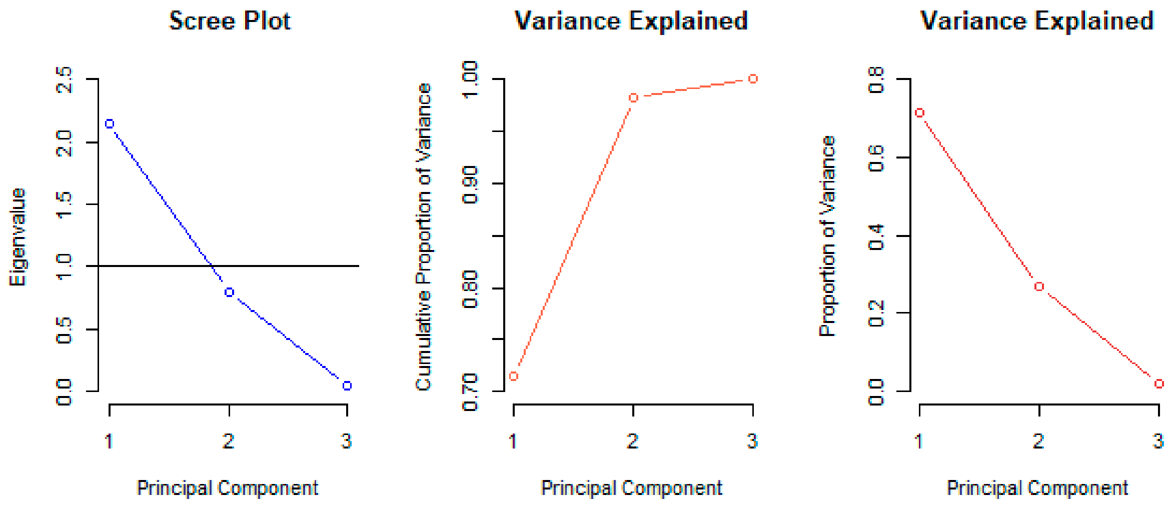

Figure S6. Scree plot representing the eigenvalues and plots with the variance accounted by the principal components (cumulative proportion of variance and proportion of variance).

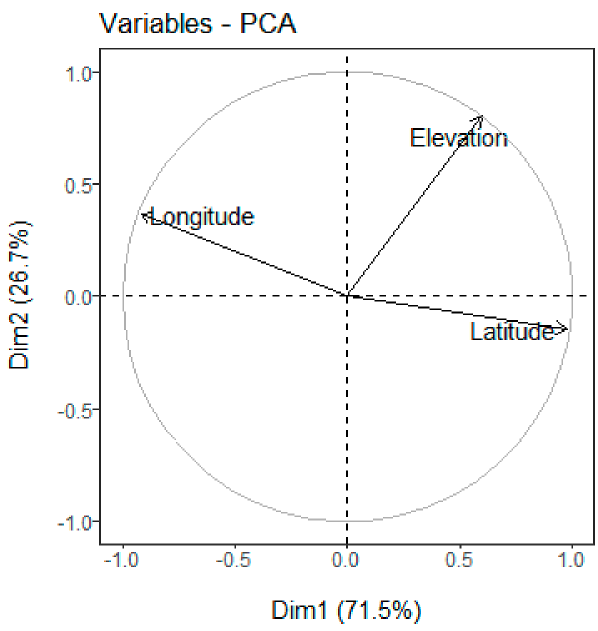

Figure S7. Graph of geographic variables from Principal component analysis (PCA). The contribution of the first two components to the total variation is denoted on each axis.

Table S13. DHARMa nonparametric dispersion test via sd of residuals fitted vs simulated to check equidispersion assumption in final model selected (GLMM fitted to Generalized Poisson distribution and log link function with crossed random effects at the *Station*, *Nest box identity*, *Female identity*, and *Year*) that assess the effects of first principal component (PC1) on litter size in the edible dormouse *Glis glis* populations across the NE of the Iberian Peninsula.

**Model**	**Explanatory variables**	**Overdispersion test**	
		**Dispersion ratio**	***p*-value**
Generalized Poisson model	PC1	1.066	0.6

Abbreviations: *PC1*, scores of the first principal component of PCA.
Note: Equidispersion occurs if the dispersion parameter approaches 1. $p < 0.05$ are marked in bold face.

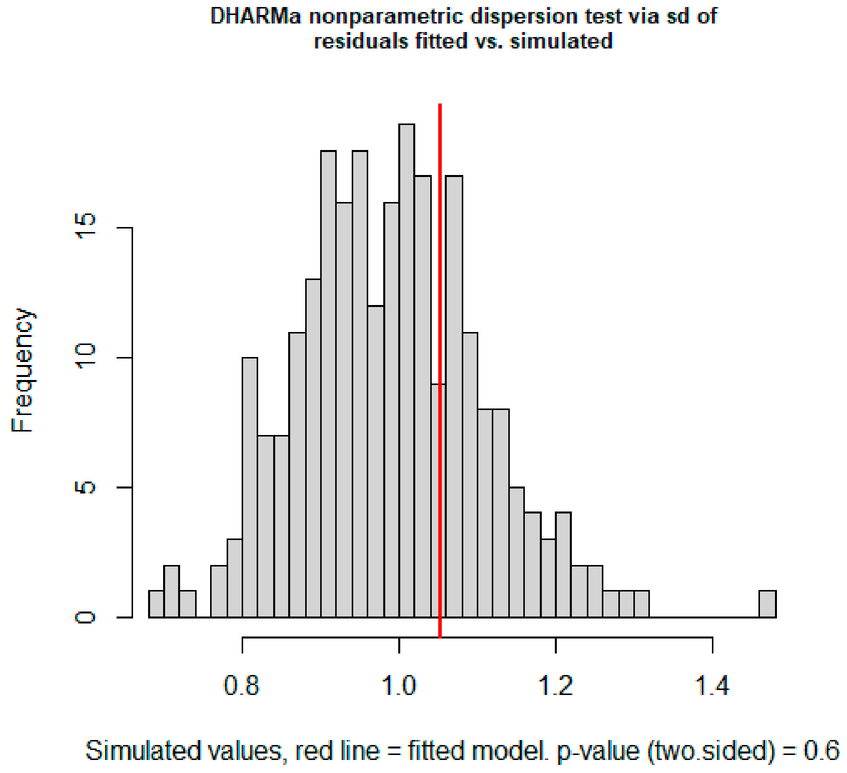

Figure S8. Plot of DHARMa nonparametric dispersion test via sd of residuals fitted vs simulated to check equidispersion assumption in final model selected (GLMM fitted to Generalized Poisson distribution and log link function with crossed random effects at the *Station*, *Nest box identity*, *Female identity*, and *Year*) that assess the effects of first principal component (PC1) scores on litter size in the edible dormouse *Glis glis* populations across the NE of the Iberian Peninsula.

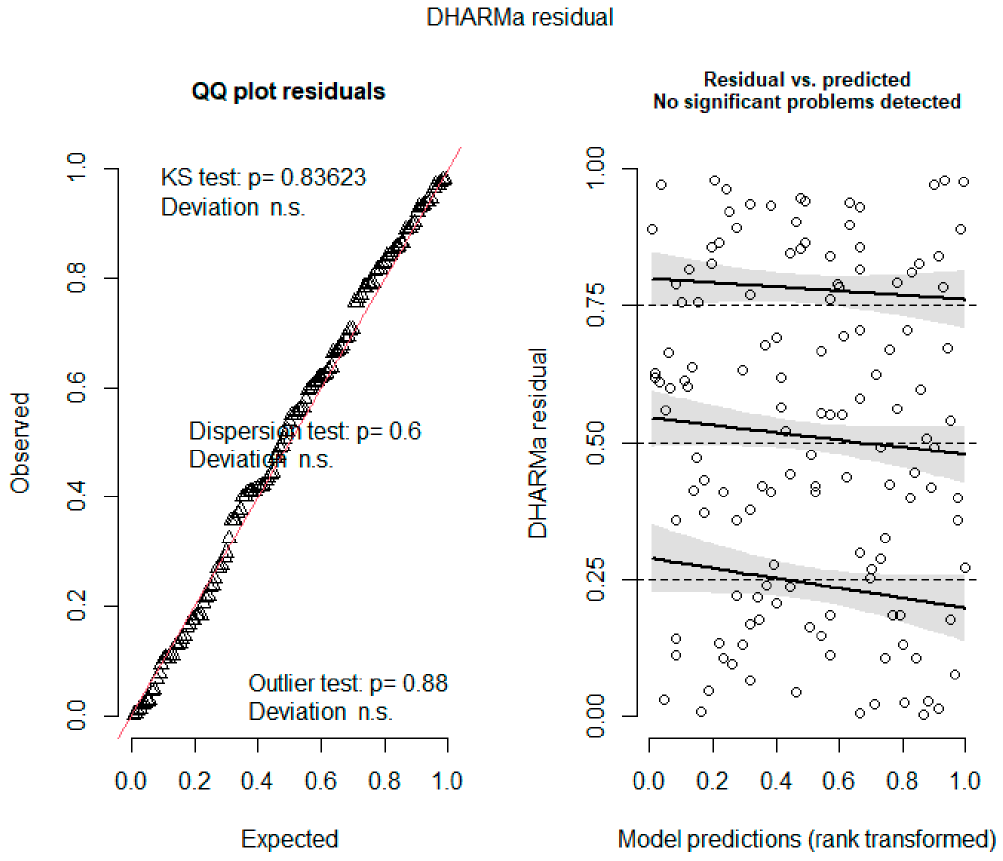

Figure S9. Residual diagnostic plots for final model selected (GLMM fitted to Generalized Poisson distribution and log link function) that assess the effects of PC1 scores on litter size in the edible dormouse *Glis glis* populations across the NE of the Iberian Peninsula. Left panel shows QQ-plot to detect deviations from the expected distribution, and right panel shows plot of the residuals against the predicted value.

References

Vekhnik, V.A. Postembryonic Development of The Edible Dormouse (*Glis glis* Linnaeus, 1766). *J. Adv. Zool.* **2022**, *43*, 32-42. http://jazindia.com/index.php/jaz/article/view/112

Gros, J.; Ligges, U. nortest: Tests for Normality. R package version 1.0-4. 2015. Available online: https://CRAN.R-project.org/package=nortest (accessed on 7 November 2022).

Fox, J.; Weisberg, S. *An R companion to applied regression*, 3rd ed.; Thousand Oaks CA: Sage, 2019. https://socialsciences.mcmaster.ca/jfox/Books/Companion/

R Core Team. *R: A Language and Environment for Statistical Computing*; version 4.0.5. R Foundation for Statistical Computing: Vienna, Austria, 2021. Available online: https://www.R-project.org (accessed on 5 July 2022).

Morris, P.A.; Morris, M.J. A 13-Year Population Study of the Edible Dormouse *Glis glis* in Britain. *Acta Theriol. (Warsz.)* **2010**, *55*, 279-288. https://doi.org/10.4098/j.at.0001-7051.066.2009

Burnham, K.P.; Anderson, D.R. *Model Selection and Multimodel Inference: A Practical Information-Theoretic Approach*, 2nd ed.; Springer: New York, NY, USA, 2002; ISBN 978-0-387-95364-9.

Maes, H.H.; Neale, M.C.; Kirkpatrick, R.M.; Kendler, K.S. Using Multimodel Inference/Model Averaging to Model Causes of Covariation between Variables in Twins. *Behav. Genet.* **2021**, *51*, 82-96. https://doi.org/10.1007/s10519-020-10026-8

Keller, I.; Alexander, J.M.; Holderegger, R.; Edwards, P.J. Widespread Phenotypic and Genetic Divergence along Altitudinal Gradients in Animals. *J. Evol. Biol.* **2013**, *26*, 2527-2543. https://doi.org/10.1111/jeb.12255

Jump, A.S.; Mátyás, C.; Peñuelas, J. The Altitude-for-Latitude Disparity in the Range Retractions of Woody Species. *Trends Ecol. Evol.* **2009**, *24*, 694-701. https://doi.org/10.1016/j.tree.2009.06.007

Bolker, B.M.; Brooks, M.E.; Clark, C.J.; Geange, S.W.; Poulsen, J.R.; Stevens, M.H.H.; White, J.S.S. Generalized Linear Mixed Models: A Practical Guide for Ecology and Evolution. *Trends Ecol. Evol.* **2009**, *24*, 127-135. https://doi.org/10.1016/j.tree.2008.10.008

Brooks, M.E.; Kristensen, K.; van Benthem, K.J.; Magnusson, A.; Berg, C.W.; Nielsen, A.; Skaug, H.J.; Mächler, M.; Bolker, B.M. glmmTMB Balances Speed and Flexibility Among Packages for Zero-inflated Generalized Linear Mixed Modeling. *R J.* **2017**, *9*, 378-400. https://doi.org/10.32614/RJ-2017-066

Lüdecke, D.; Ben-Shachar, M.; Patil, I.; Waggoner, P.; Makowski, D. performance: An R Package for Assessment, Comparison and Testing of Statistical Models. *J. Open Source Softw.* **2021**, *6*, 3139. https://doi.org/10.21105/joss.03139

Brooks, M.E.; Kristensen, K.; Darrigo, M.R.; Rubim, P.; Uriarte, M.; Bruna, E.; Bolker, B.M. Statistical Modeling of Patterns in Annual Reproductive Rates. *Ecology* **2019**, *100*, 1-7. https://doi.org/10.1002/ecy.2706

Kaiser, H.F. The Application of Electronic Computers to Factor Analysis. *Educ. Psychol. Meas.* **1960**, *20*, 141-151. https://doi.org/10.1177/001316446002000116

Kassambara, A.; Mundt, F. factoextra: Extract and Visualize the Results of Multivariate Data. R Package Version 1.0.7. 2020. Available online: https://CRAN.R-project.org/package=factoextra (accessed on 10 December 2022).

Hartig, F. DHARMa: Residual Diagnostics for Hierarchical (Multi-Level/Mixed) Regression Models. R Package Version 0.4.5. 2022. Available online: https://cran.r-project.org/package=DHARMa (accessed on 10 December 2022).
